# Pathways of Hypoxia-Inducible Factor (HIF) in the Orchestration of Uterine Fibroids Development

**DOI:** 10.3390/life13081740

**Published:** 2023-08-13

**Authors:** Maria Fedotova, Ekaterina Barysheva, Olga Bushueva

**Affiliations:** 1 Laboratory of Genomic Research, Research Institute for Genetic and Molecular Epidemiology, Kursk State Medical University, 305041 Kursk, Russia; 2 Department of Biology, Medical Genetics and Ecology, Kursk State Medical University, 305041 Kursk, Russia

**Keywords:** uterine fibroids, uterine myoma, leiomyoma, hypoxia, hypoxia-inducible factor, HIF, HIF-1 signaling pathway, review, gene expression, protein–protein interactions

## Abstract

Uterine fibroids (UF) are common benign tumors in women. The course of UF is associated with troubling symptoms and the development of infertility and pregnancy pathology. Surgical treatment even implies hysterectomy, while pharmacological interventions are modestly effective. Classically, hypoxic metabolism is considered a hallmark of malignant tumor. However, the role of hypoxia-induced factor (HIF) is significant in benign tumors as well. Herein, we briefly review the basic biology of HIF-family proteins, outlining their possible roles in UF. Apart from theoretical justifications, we summarized 15 studies reporting increased expression of HIFs and downstream factors in UF samples. Altogether, data suggest that increased expression of the HIF-protein and altered expression of its dependent genes are presumed to be the factors leading to UF development. Thus, even without being a malignant tumor, UF is characterized by the strong involvement of HIF. This novel insight may give rise to further research in the direction of finding new prognostic markers and effective medicines against UF.

## 1. Introduction

Uterine fibroids (UF), also known as uterine myoma or uterine leiomyoma, are the widespread benign tumors of the female reproductive system, affecting approximately 70% of white women and 80% of women of African descent during their lifetime [1]. In approximately 30% of cases, UF manifest with abnormal uterine bleeding, pelvic pain, pressure, or discomfort, as well as anemia. Whereas malignant transformation of UF is a relatively rare phenomenon [2], the conditions associated with fibroids are still severe and might include infertility. Moreover, whereas UF could be the only cause of infertility in 2–3% of cases [3], these neoplasms cause complications during pregnancy and delivery [4]. Unfortunately, the management of patients with UF frequently relies on surgical approaches, potentially resulting in hysterectomy, which is one of the worst scenarios for women desiring future pregnancy. Moreover, while surgical interventions may decrease the chances of a healthy pregnancy, current pharmacological approaches demonstrate modest efficacy or serious adverse effects. For instance, currently available medications modulating different levels of the hypothalamic–pituitary–gonadal axis provide only short-term relief [5] and may cause adverse effects—bone loss [6] and a wide spectrum of other effects [7]. Additional pharmacological options such as nonsteroidal anti-inflammatory drugs and tranexamic acid help to reduce blood loss, but they do not have any proven effect on tumoral growth.

Obviously, further studies of the molecular pathways underlying UF development and malignant transformation may provide novel insights into more effective and safe therapies. In this regard, herein, we review the role of crosstalk between molecular pathways driving UF and HIFs, the family of well-known proteins orchestrating cellular oxygen sensing and response to hypoxia.

## 2. Methodology

Hypothesizing the role of HIF pathways in UF, we performed a literature analysis via PubMed and Google Scholar to identify relevant studies disclosing the role of HIF-proteins in benign myometrium/leiomyoma tumors. Outlining basic evidences bridging HIF and UF we also extracted studies revealing the contribution of HIF-responsive genes/proteins (the list was found in the Kegg pathway database). Combinations of the key words were as follows: uterine fibroids, leiomyoma, myoma, expression, names of chosen genes/proteins: HIF, *ANGPT1*, VEGF, *FLT1*, *EGF*, *PAI-1*, SEPRINE1, *TIE-2*, *TEK*, TIMP-1, Endothelin-1, *EDN1*, iNOS, eNOS, *ANP*, *EPO*, *TF*, *HMOX1*, *TFRC*, GLUT-1, *SLC2A1*, PDK-1, HK, *PFKL*, *GAPDH*, ALDOA, ENO1, PGK, *PFK2*, *LDHA*, Bcl-2, p21, cyclin-dependent kinase inhibitor 1A, and *CD18*. Bibliographies were cross-referenced to identify additional studies. All relevant articles and additional articles cited in primary references are included. To search for genes associated with the risk of UF at the whole-genome level of significance, we used the GWAS catalog (https://www.ebi.ac.uk/gwas/ (accessed on 1 June 2023)). Then, the STRING database online bioinformatics tool (https://string-db.org/ (accessed on 3 June 2023)) was used to determine which GWAS genes are in direct interaction with the genes encoding HIF subunits. The STRING database contains information about mechanisms of protein–protein association within a common framework. The search for common biological processes (the so-called overrepresented biological processes) involving the HIF genes and the GWAS genes directly interacting with them was carried out using the Gene Ontology online tool (http://geneontology.org/ (accessed on 4 June 2023)).

## 3. UF in Terms of Hypoxia Response

The benign nature of UF suggests that the cells maintain the main characteristics of normal myocytes. However, they are also distinguished by specific features. In tumors, metabolism always shifts to a hypoxic state due to two factors. The first one is that even under normoxic conditions, tumor cells fail to utilize pyruvate in the tricarbonic acids cycle but use it to produce lactate. This is one of the most typical phenomena called the Warburg effect [8]. The mechanisms underlying this effect have yet to be understood in detail, but it allows tumor cells to benefit by accelerating anabolic processes [9]. The second factor is that the rapid growth of the tumor cells overtakes its vascularization, causing imbalanced feeding and insufficient blood supply.

As a result, metabolic products are accumulated and the cells send neighbors “hypoxic” help signaling, which increases vascularization, Fe-accumulation, glucose uptake and inflammatory response [10]. Hypoxic signaling also affects cells of normal myometrium (NM), endowing them with traits inherent in UF such as aberrant cell cycle dynamics or abnormal metabolism [11]. UF is characterized by high oxidative stress due to impaired redox metabolism [12] and increased reactive oxygen species production in response to hypoxia [13]. Moreover, when the metabolism of UF is switched to the direction of hypoxia, the tumor obviously increases its chances of malignant transformation.

## 4. Basic Biology of HIF

Since aerobic metabolism is a universal sign of all *Metazoa*, the identification of molecular sensors of oxygen can be called one of the most important discoveries in the last 30 years [14]. 

This origin of this discovery goes back to the late 1970s with the identification of a hypoxia-responsive element of human *EPO* 3′ enchancer in liver and renal cells [15]. It was suggested that this specific oxygen-sensing mechanism is specific for EPO-producing cells. However, further studies revealed hypoxia-responsive element-binding activity in tissues not expressing EPO: lungs, aortic endothelium, etc. [16]. The next landmark was the identification of the main sensor of hypoxia, the HIF itself [17], as well as upstream and downstream members of its pathway: the complex of von Hippel–Lindau (VHL)–Elongin B and C-CUL2 [18,19]. Vascular endothelial growth factor (VEGF) [20], the prolyl-hydroxylase family [21], and factor inhibiting HIF [22,23].

There are three isoforms of HIF proteins, each consisting of alpha (α) and beta (β) subunits. HIF-1 and HIF-2 are the most studied members, characterized by similar protein structures. Notably, HIF-1 is a master regulator of the acute response to oxygen depletion, whereas HIF-2 is responsible for adaptation to prolonged hypoxia. Molecular biology and the particular role of HIF-3 have yet to be studied in detail; however, it regulates various pathways, including HIF-1 and HIF-2 [24,25].

The alpha subunit of each isoform is an inducible part normally located in the cytoplasm. The second one, beta, is a constitutively expressed part located in the nucleus. During normoxia, prolyl hydroxylases add hydroxyl residues to the α-subunit, making it a target for ubiquitin-dependent breakdown via proteasome degradation. Another regulator of HIF activity is the factor inhibiting HIF, which adds hydroxyl residues to the asparagine and thus interferes with the binding of HIF and transcriptional coactivators. Since both factors function in an oxygen-dependent manner, they are unfunctional during hypoxia. Thus, as one’s oxygen level decreases, factors inhibiting HIF and prolyl hydroxylase domain proteins are inhibited, enabling the alpha subunit to be stabilized with further translocation into the nucleus and assembly with the beta subunit. The formed HIF heterodimeric complex binds to the promoter of HIF-responsive genes in order to modulate their expression [26].

## 5. The Role of Hypoxia-Inducible Factor in Uterine Fibroids

In keeping with its essential physiological role, HIF-1 has been demonstrated to contribute in a widest spectrum of human pathologies, from oncological to neurodegenerative and infectious [15]. Not surprisingly, HIF-1 is also strongly involved in various gynecological diseases—preeclampsia [27], polycystic ovary syndrome [28], cancer [29], endometriosis [30] and others [31,32].

Several studies have expectedly reported increased HIF1-α expression in UF [33,34,35]. In order to further elucidate this link, we carried out an analysis of available data related to expression of genes involved in the HIF-1 signaling pathway in UF based on the KEGG pathway database. It is known that the HIF pathway includes many genes with major effects on the processes typical for UF pathogenesis: apoptosis, proliferation, metabolism, regulation of the cellular availability of oxygen and nutrients [36]. In particular, such proteins as VEGF-A, ANG-1 (angiopoietin-1), VEGFR-1, EGF, SERPINE1 (PAI1), TEK, and TIMP1 regulate the division, proliferation, and motility of vascular cells as well as the disorganization of the extracellular matrix, thus favoring angiogenesis [37]. Factors like HMOX1, EDN1, iNOS, and cNOS provide control of vascular tone by balancing nutrients and oxygen supply [38]. Additionally, NO-synthases are involved in the regulation of apoptosis in UF cells by nytrosylation of caspase 3, downregulating Caspase 3 activity, and increasing the Bcl2/Bax ratio [39]. Finally, the HIF-dependent response to oxygen depletion is critically dependent on the glucose carrier protein GLUT-1 and glycolytic enzymes: HK, ALDOA, ENO1, PGK1, PFK/FBPase 3, and LDHA [40] (Table 1).

## 6. HIF and GWAS-Identified Genes

**Figure 1:**
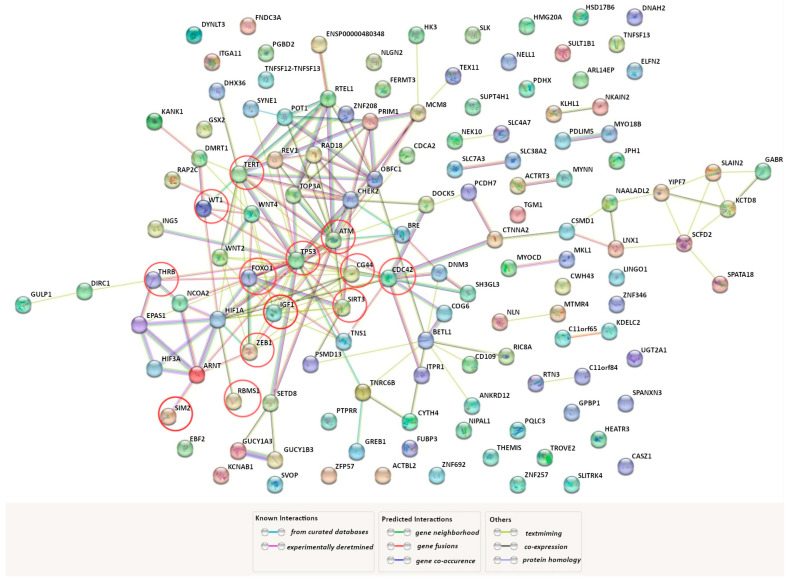
Predicted functional partners of genes encoding HIF subunits across the genes associated with UF (https://string-db.org/ (accessed on 3 June 2023)). A network of protein–protein interactions among the genes encoding HIF subunits (HIF1A, HIF1B (ARNT), HIF2A (EPAS1), and HIF3A).and genes identified as a result of genome-wide association studies (GWAS) of UF. The factors, directly (without secondary mediators) interacting with the HIF subunits, are marked with red circles. PPI enrichment *p*-value: <1.0 × 10^−16^.

GWAS remains one of the most effective approaches to the search for “susceptibility” genes to human multifactorial diseases [69,70,71,72]. Using the GWAS catalog, we found that variations in 174 genes are associated with UF risk in different populations around the world (for instance, rs16991615 *MCM8*, rs17631680 *DNMT3AP1*, rs2306022 *ITGA11*, rs2456181 *ZNF346*, rs3820282 *WNT4*, rs4335411 *ZNF692*, rs5930554 *AGKP2*, rs66998222 *HNRNPA1P48*, rs78378222 *TP53*, rs7986407 *FOXO1*, rs8105767 *ZNF257*, and rs9548898 *COG6*). We found it interesting to analyze how genes that encode HIF subunits (*HIF1A*, *HIF1B* (*ARNT*), *HIF2A* (*EPAS1*), *HIF3A*, *HIF2B*, *HIF3B*) interact with those GWAS-selected genes in a functional way. For this purpose, we have used the bioinformatics tool STRING (https://string-db.org/ (accessed on 3 June 2023)). The interactome network of protein–protein interactions (PPI) obtained using the source is shown in Figure 1. HIF2B and HIF3B were not included in the analysis of the PPI network since the STRING database does not provide data on them. It turned out that 72 GWAS genes, directly or indirectly through other functional partners, are involved in the interaction with *HIF1A*, *HIF1B*, *HIF2A*, and *HIF3A*. During analysis of the STRING network, we identified 13 GWAS genes that directly interact with genes encoding HIF subunits: *ATM*, *CD44*, *CDC42*, *FOXO1*, *IGF1*, *RBMS1*, *SIM2*, *SIRT3*, *TERT*, *THRB*, *TP53*, *WT1*, *ZEB1* (marked with red circles in Figure 1). 

Together with *HIF1A*, *HIF1B*, *HIF2A*, and *HIF3A*, these genes jointly participate in a large number of pathogenetically significant UF biological processes, characterizing hypoxia, oxidative stress apoptosis, proliferation, cell growth, adhesion and migration, myoblast differentiation, metabolic changes, morphogenesis of vessels, and others (Table 2). In particular, we would like to note the participation of this set of genes in overrepresented biological processes involved in the mechanisms of cellular reply in response to hypoxia and oxidative stress; in the regulation of apoptosis directly and through the P53 signaling; in the regulation of vascular homeostasis through nitric-oxide synthase activity; participation in neoangiogenesis through the regulation of vascular associated smooth muscle cell proliferation; as well as participation in the processes that directly control the initiation and progression of the tumor: “myoblast differentiation”, “positive regulation of cell growth, “mesenchymal cell differentiation”.

Taking into account the fact that these processes are reflectors of high-functional HIFs’ interaction with genes, established as a result of human genome-wide association studies (GWAS), this provides conclusive evidence of the role of the HIF pathway genes we have studied in key molecular determinants of tumor growth.

**Figure 2:**
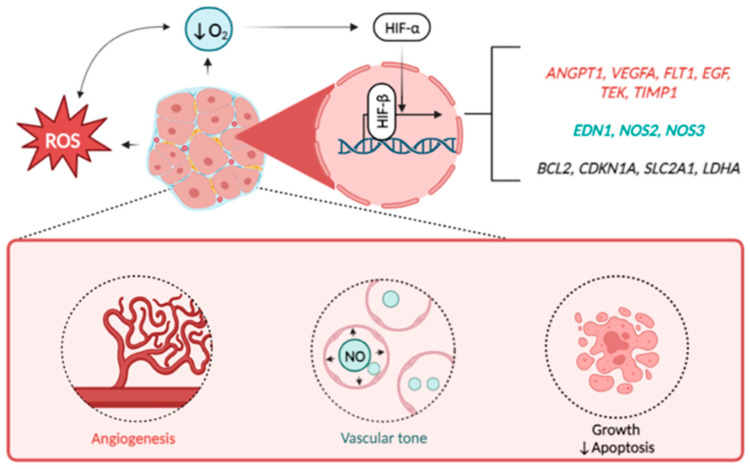
HIF-downstream signaling in the orchestration of UF development. Note: Excessive production of reactive oxygen species (ROS) and decreased oxygen supply induce the activation of HIF alpha subunits (mostly HIF-1a) which heads to the nucleus and binds to beta subunit. Interacting, both subunits launch the transcription of wide spectrum of HIF-responsive genes of hypoxia response. Besides the others these genes include regulators of vascular growth (*ANGPT1*, *VEGFA, FLT1, EGF, TEK, TIMP1*), vascular tone (*EDN1, NOS2, NOS3*), apoptosis (*BCL2*), cell cycle (*CDKN1A*), as well as glucose transporter GLUT1 (*SLC2A1*) and lactate dehydrogenase (*LDHA*). Altogether, these factors promote the growth rate of UF thus playing negative role in disease pathogenesis.

Altogether, the summarized data reflecting previous research and our bioinformatic findings are interpretated in Figure 2.

## 7. Future Perspectives

Although the presented data provide a solid ground to bridge HIF and pathogenesis of UF, there is no rationale to distinguish whether HIF is involved in growth, onset, or both. As upregulated levels of HIF and its downstream effectors were detected in UF samples, HIF is involved in progression of the disease. However, future studies can help to elucidate its role in etiology of the disease.

Further investigation can also focus on identifying specific markers associated with HIF expression and downstream factors in UF. Understanding these markers could aid in predicting disease progression, treatment response, and potential complications. The recognition of HIF involvement in UF suggests that targeting HIF pathways could be a potential therapeutic strategy. Developing medications that effectively modulate HIF expression or its downstream factors may offer improved treatment options for UF patients. Further research are needed to elucidate the complete mechanisms by which HIF influences UF development. This sort of research includes studying the interactions between HIF and other molecular pathways implicated in UF pathogenesis, such as hormonal factors, epigenetic regulation and immune responses. Obtaining a comprehensive understanding of the complex biology underlying UF will contribute to improved diagnostic and therapeutic strategies.

In recent decades, there has been a growing interest in the study of HIF and its targets in oncology. This attention is of highest clinical significance first of all because of the opportunity to develop new pharmacological avenues for the treatment of various pathological conditions, first of all cancer [73]. In this respect, one may observe a number of HIF-targeted medications undergoing clinical trials in cancer patients. Most HIF inhibitors are characterized by suppressive activity against cancer. For instance, PX-478 promotes apoptosis, suppresses tumor proliferation, epithelial-mesenchymal transition, and arrests the cell cycle in G2 phase, thus inhibiting tumor growth [74].

There are also several HIF-2a inhibitors undergoing clinical studies. Results of completed phase II clinical trials of HIF-2a inhibitor PT2385 in treating patients suffered from recurrent glioblastoma were posted in 2020 (https://beta.clinicaltrials.gov/study/NCT03216499?tab=history (accessed on 10 June 2023)). A few clinical trials of HIF-2α inhibitors were started in 2021. NKT2152 is being studied in a phase I/II clinical trial in patients suffering from advanced clear-cell renal cell carcinoma. https://clinicaltrials.gov/ct2/show/study/NCT05119335?term=HIF+inhibitor&cond=cancer&draw=2&rank=4 (accessed on 10 June 2023) [75].

DFF332, Another HIF2-α inhibitor for patients with renal cell carcinoma and other tumors (such as HLRCC and VHL disease), is already undergoing phase I/Ib (https://clinicaltrials.gov/ct2/show/NCT04895748?term=DFF332&draw=2&rank=1 (accessed on 10 June 2023)).

Such a wide range of pharmacological tools gives a reason to hope for near-term applications targeting HIF in UF patients. Further studies can help to evaluate the long-term effects of HIF-suppressing therapy in order to find the most efficient and safest drugs. In this respect, one may probably expect the appearance of a rationale to use some of the mentioned medicines to treat UF. Our review gave a good reason to consider HIF-suppressing pathways to decelerate the growth rates of the UF. In this respect, potential application of HIF-inhibitors may serve a new avenue to prevent some of complications related to the disease. For instance, apart from bleeding UF can potentially cause infertility by blocking the fallopian tubes or stopping a fertilized egg from implanting in the uterus [76]. If the inhibition of HIF turns out successful therapy, women with poor fertility prognosis, miscarriages or complicated pregnancies in anamnesis would may be prescribed a new type of nonsurgical interventions to be treated. Noteworthy, that anatomical features of UF allow topical application of the medical compounds thus minimizing systemic side effects. Thus, we are assuming that the selective and local inhibition of HIF pathways may serve a novel approach to the treatment of UF.

## 8. Limitations

This work was based on the literature review and did not imply following classical principles of meta-analysis such as assessing of statistical power and strict inclusion/exclusion criteria. The authors admit the limitations related to a low number of studies directly evaluating the role of HIF protein in UF. All the theoretical justifications require experimental approval.

## 9. Conclusions

This article has demonstrated for the first time a growing understanding of molecular and genetic determinants of UF development, orchestrated by HIF. Moreover, as far as we are aware, this work is the first review emphasizing the role of HIF in benign tumors. The HIF pathway activates expression of a number of genes responsible for processes known to be imbalanced in tumors. Analysis of patterns of mRNA and protein expression of HIF-target genes, which play a key role in UF development, showed that UF cells displayed their altered status. Additionally, we revealed that genes encoding HIF subunits interact with genes associated with the development of UF in a functional way. Thirteen proteins encoded by those genes are directly interacting with HIFs: Additionally, we distinguished the main biological processes, provided by the revealed genes. These processes cover the whole spectrum of the UF-driving mechanisms: proliferation, cell growth, adhesion and migration, myoblast differentiation, metabolic changes, morphogenesis of vessels, and others. Summarizing the considerable role of hypoxia and HIF in UF pathogenesis, we expect that further studies of this disease narrowly focused on HIF-related pathways may bring new insight on more effective therapeutic options.

## Figures and Tables

**Table 1:** Main HIF-responsive factors, imbalanced in UF.

Factor *	Function **	Reference	Design of the Study	Main Findings in UF
VEGF-A	Promotes the PI3K/Akt pathway, endothelial cell growth, and neo-angiogenesis.	Hague et al. [41]	Comparison of VEGF-A protein levels between women with UF (*n* = 52) and women without UF (control group, *n* = 39).	Increased VEGF-A expression was detected in UF compared with controls (*p* < 0.05).
		Korompelis et al. [42]	Comparison of the blood levels of VEGF-A protein in women with UF (*n* = 46) and healthy controls (*n* = 39).	VEGF-A was significantly elevated in the blood of UF patients compared with controls (*p* < 0.001).
		Wei et al. [43]	Comparison of VEGF protein in UF tissue (*n* = 7) and normal myometrium from the same patients (*n* = 7) Measurements were performed in six zones of UF, from the periphery to the centre.	The level of hypoxia has a linear correlation to VEGF expression levels. VEGF concentrations in UF were gradually increasing from the periphery to the central UF zone (*p* < 0.05).
		Asano et al. [44]	Comparison of VEGF mRNA in MED12-mutated UF (*n* = 52) and MED12-wild-type UF (*n* = 56).	MED12-mutated UF expressed higher VEGF mRNA levels compared with wild-type tumors (*p* = 0.024).
		Joo et al. [45]	Comparison of VEGF-A mRNA levels in intramural (*n* = 19) and subserosal (*n* = 4) UF tissue and normal myometrium (*n* = 10).	VEGF-A mRNA was significantly higher in both UF types (*p* < 0.05).
FLT-1 (VEGFR-1)	Encodes vascular endothelial growth factor receptor 1, which mediates VEGF signaling.	Sanci et al. [46]	Comparison of FLT-1 protein between cellular UF tissue (*n* = 20) and ordinary UF (*n* = 20).	Moderate FLT-1 immunostaining intensities in UF and its higher level in cellular UF compared with ordinary UF (*p* < 0.01).
EGF	Induces angiogenesis, cell growth, and differentiation through binding with EGFR.	Tsiligiannis et al. [47]	The level of EGF mRNA was compared between UF (*n* = 11) and surrounding normal myometrial tissue (*n* = 11).	An increased level of EGF expression in UF than in NM (*p* = 0.01–0.05).
		Dixon et al. [48]	The level of EGF protein expression was studied in UF samples (*n* = 7) and matched myometrial samples (*n* = 7).	Lower EGF expression in UF than in myometrium (*p* < 0.05).
		Park et al. [49]	The level of EGF protein was compared between UF (*n* = 12) and surrounding normal myometrial tissue (*n* = 12).	There is no difference in EGF expression between UF and NM (*p* > 0.05).
SERPINE1	Inhibits two ferments: tissue plasminogen activator and urokinase, and hence fibrinolysis.	Sourla et al. [50]	Comparison of SERPINE1 mRNA between UF samples (*n* = 16) and adjacent myometrium (*n* = 16).	11 UF showed higher expression of SERPINE1 mRNA compared with the adjacent myometrium, and 5 UF showed a lower level (*p*< 0.05).
		Cheng et al. [51]	Comparison of SERPINE1 mRNA and protein in UF samples (*n* = 30) and the adjacent myometrial tissue.	Higher expression of SERPINE1 mRNA and protein was investigated in UF compared with the adjacent myometrium (*p* < 0.05).
TEK	Angiopoietin-1 receptor. Participates in angiogenesis.	Tsiligiannis et al. [9]	Comparison of TEK mRNA expression between UF (*n* = 11) and surrounding normal myometrial tissue (*n* = 11).	Decreased level of TEK expression in UF compared with NM (*p* < 0.01).
TIMP1	Inhibits proteins from the MMP family. It takes part in extracellular matrix restructuring and slows down endothelial cell migration.	Bogusiewicz et al. [52]	Comparison of TIMP1 protein in UF (*n* = 20) and the corresponding myometrium (*n* = 20) of hysterectomized women.	There are no differences between comparable groups (*p* > 0.05).
		Tsiligiannis et al. [47]	Comparison of TIMP1 mRNA between UF (*n* = 11) and surrounding normal myometrial tissue (*n* = 11).	Higher level of TIMP1 mRNA in UF and proximal myometrium compared with distal myometrium (*p* > 0.1–0.2).
		Korompelis et al. [42]	Comparison of blood TIMP1 protein level in women with UF (*n* = 46) and healthy controls (*n* = 39).	TIMP1 blood level was elevated in UF patients compared with healthy woman (*p* = 0.003).
		Governini et al. [53]	Comparison of TIMP1 mRNA in women with UF (*n* = 18) and control women (*n* = 18).	There were no differences in TIMP1 expression (*p* > 0.05).
EDN1	Strong activator of vasoconstriction.	Pekonen et al. [54]	Comparison of EDN1 mRNA levels in UF (*n* = 8) and adjacent myometrium (*n* = 8).	There is no difference between normal myometrium and UF in the abundance of EDN1 mRNA (*p* > 0.05).
		Wallace et al. [55]	Comparison of EDN1 protein blood levels in UF (*n* = 32) and the control group (*n* = 11). The secretion of ET-1 protein under hypoxia and normoxia was studied using UF samples (*n* = 32), samples from the adjacent myometrium (*n* = 32), and samples from control women (*n* = 11). EDN1 transcript-preproendothelin mRNA (PPET) was quantitated in UF (*n* = 7) and NM (*n* = 7) under normoxic and hypoxic conditions.	The circulating EDN1 level was greater in UF patients compared with controls (*p* < 0.005). Secretion of EDN1 was higher in UF compared with adjacent myometrium (*p* = 0.025). Hypoxia-induced ET-1 secretion from UF was higher compared to normoxic UF (*p* = 0.001). EDN1 secretion from UF cultured under normoxia was higher compared with adjacent myometrium (*p* = 0.02). EDN1 secretion was higher in UF exposed to 24 h of hypoxia (*p* = 0.005) compared with myometrium explants exposed to hypoxia. Under normoxia, PPET mRNA was increased in UF compared with NM (*p* = 0.01) and in hypoxic UF compared to hypoxic NM (*p* = 0.002).
		Miyashita-Ishiwata et al. [33]	Comparison of EDN1 in UF and myometrial cells cultured under normoxic and hypoxic conditions.	Hypoxia induced EDN1 expression in the culture media in leiomyoma but not in myometrial cells.
iNOS	Inducible enzyme, which produces NO.	Plewka et al. [56]	Comparison of iNOS protein levels in the myometrium of six groups of women with different myoma sizes in perimenopausal and reproductive age.	Increased NO expression in both small and large UF compared to control in women of reproductive and perimenopausal age (*p* = 0.05). Higher iNOS expression in large myomas compared to small ones (*p* = 0.05).
		Fletcher et al. [39]	Comparison of expression of iNOS mRNA and nitrate/nitrite ratio (activity index of iNOS) in UF and NM cell lines exposed to normal (20% O_2_) and hypoxic (2% O_2_) conditions for 24 h.	Higher iNOS mRNA expression (*p* = 0.059) and total nitrate/nitrite ratio (*p* < 0.04) in UF than in NM under normoxic conditions. Hypoxic conditions led to a more significant increase in iNOS levels in NM (*p* < 0.001) than in UF (*p* = 0.268). Under hypoxia, the nitrate/nitrite ratio increased in UF (*p* = 0.091) and NM (*p* < 0.001).
cNOS (eNOS)	Produces NO, the predominant isoform in endothelial cells.	Oh et al. [57]	Comparison of cNOS protein in two groups of perimenopausal women with UF (*n* = 24) and the control group of healthy patients (*n* = 8).	Higher level of cNOS expression in the UF women compared with the control (*p* = 0.05). Inside UF patients, cNOS expression was higher in symptomatic patients (menorrhagia and dysmenorrhea) compared to asymptomatic patients (endometrium *p* = 0.0029, myometrium *p* = 0.0276).
		Gokdeniz et al. [58]	Comparison of cNOS protein levels in UF tissue and normal myometrium (*n* = 8).	Higher level of cNOS in the smooth muscle cells of UF compared to NM (*p* < 0.005).
		Joo et al. [45]	Comparison of cNOS mRNA expression in UF patients (*n* = 23) and normal myometrium (*n* = 10).	Expressions of cNOS mRNA were higher in intramural and subserosal UF compared with normal myometrium (*p* < 0.05). Expression of cNOS mRNA was higher in large UF than small UF (*p* < 0.05).
EPO	Activator of erythropoiesis in the bone marrow. Under hypoxic conditions, it increases the oxygen-carrying capacity of the blood.	Asano et al. [59]	Comparison of EPO mRNA expression in UF patients (*n* = 114) and NM from these patients (*n* = 17).	Higher mRNA expression of EPO in the UF than in the NM (*p* = 0.025).
		Asano et al. [44]	Comparison of EPO mRNA expression in MED12-mutated UF (*n* = 52) and MED12-wild-type UF (*n* = 56).	The EPO mRNA level was threefold higher in UF with wild-type MED12 genes compared to mutated ones (*p* = 0.01).
GLUT-1	One of the glucose transporter families. Activates glucose passing through the blood–brain barrier, the cell membranes of red blood cells, and tumors.	Ishikawa et al. [35]	Comparison of GLUT-1 mRNA expression in cell cultures of UF under hypoxia and normoxia.	Hypoxia induced the GLUT-1 mRNA expression (*p* < 0.05).
		Knapp et al. [60]	Comparison of GLUT-1 mRNA expression in NM specimens and UF women (*n* = 74).	Higher GLUT-1 mRNA expression in UF compared to NM (*p* < 0.05).
HK1	The first enzyme of glycolysis, phosphorylating glucose to glucose-6-phosphate.	Kwon et al. [61]	Comparison of HK1 mRNA derived from UF and corresponding NM were labeled with Cy5 and Cy3 fluorescein (*n* = 5).	HK1 mRNA was higher in UF compared to NM (*p* < 0.05).
		Catherino et al. [62]	Comparison of 3 UF and NM tissue pairs obtained from a patient with the HLRCC (hereditary leiomyomatosis and renal cell cancer) and patients with nonsyndromic or common UF (*n* = 11).	Similar expression of HK1 mRNA in nonsyndromic UF and HLRCC compared to NM (*p* > 0.05).
ALDOA	Glycolytic enzyme which provides the reversible conversion of fructose-1,6-bisphosphate to dihydroxyacetone phosphate and glyceraldehyde 3-phosphate.	Ishikawa et al. [35]	Comparison of mRNA expression of ALDOA in cells cultures of UF under hypoxia and normoxia.	Hypoxia induced the ALDOA mRNA expression (*p* < 0.05).
		Catherino et al. [62]	Comparison of 3 UF and NM tissue pairs obtained from a patient with the HLRCC (hereditary leiomyomatosis and renal cell cancer) and patients with nonsyndromic or common UF (*n* = 11).	HLRCC fibroids overexpressed ALDOA mRNA (*p* < 0.05). Expression of this gene was not changed in nonsyndromic UF (*p* < 0.01).
ENO1	Glycolytic enzyme, which provides the conversion of 2-phosphoglycerate to phosphoenolpyruvate. This protein involved in allergic reaction, growth and hypoxia tolerance	Vanharanta et al. [63]	Comparison of ENO1 mRNA levels in UF carrying FH mutations (*n* = 7) and wild-type FH (*n* = 15).	Overexpression of ENO1 mRNA in FH-mutated tumors compared to nonmutated UF (*p* < 0.05).
		Catherino et al. [62]	Comparison of 3 UF and NM tissue pairs obtained from a patient with HLRCC (hereditary leiomyomatosis and renal cell cancer) and patients with nonsyndromic UF (*n* = 11).	HLRCC fibroids overexpressed ENO1 mRNA (*p* < 0.01). Expression of this gene was not changed in nonsyndromic UF.
		Ishikawa et al. [35]	Comparison of ENO1 mRNA expression in cell cultures of UF under hypoxia and normoxia.	Hypoxia induced ENO1 mRNA expression (*p* < 0.05).
PGK1	Glycolytic enzyme catalysing the conversion of 1,3-diphosphoglycerate to 3-phosphoglycerate.	Catherino et al. [62]	Comparison of 3 UF and NM tissue pairs obtained from a patient with the HLRCC (hereditary leiomyomatosis and renal cell cancer) and patients with nonsyndromic or common UF (*n* = 11).	HLRCC fibroids overexpressed PGK1 mRNA (*p* < 0.01). Expression of this gene was not changed in nonsyndromic UF.
PFK/FBPase 3	Involved in the synthesis of fructose-2,6-bisphosphate (F2,6BP) and the degradation of F2,6BP.	Catherino et al. [62]	Comparison of 3 UF and NM tissue samples obtained from patients with hereditary leiomyomatosis and renal cell cancer and patients with nonsyndromic or common UF (*n* = 11).	HLRCC fibroids overexpressed PFK/FBPase 3 mRNA (*p* < 0.01). Expression of this gene was not changed in nonsyndromic UF.
		Ishikawa et al. [35]	A comparison of PFK/FBPase 3 mRNA expression was evaluated in cell cultures of UF under hypoxia and normoxia.	Hypoxia induced PFK/FBPase 3 mRNA expression (*p* < 0.05).
LDHA	The final glycolytic enzyme that converts L-lactate to pyruvate.	Vanharanta et al. [63]	Comparison of LDHA mRNA expression in UF carrying FH mutations (*n* = 7) and UF with a wild-type FH (*n* = 15).	Overexpression of LDHA mRNA in FH-mutated tumors compared to nonmutated UF (*p* < 0.05).
		Ishikawa et al. [35]	Comparison of LDHA mRNA expression in cell cultures of UF under hypoxia and normoxia.	Hypoxia induced LDHA mRNA expression (*p* < 0.05).
Bcl-2	Antiapoptotic gene from the B-cell lymphoma gene family.	Zhang et al. [64]	Comparison of Bcl-2 protein expression in patients with UF (*n* = 34), uterine leiomyosarcoma (*n* = 34) and normal myometrial samples (*n* = 34).	Higher Bcl-2 expression in UF than in NM and in uterine leiomyosarcoma (*p* < 0.01).
		Wu et al. [65]	Comparison of Bcl-2 protein levels in UF tissue (*n* = 24) and correspondent NM (*n* = 22) in patients with UF.	Bcl-2 was overexpressed in UF compared to NM only in the proliferative phase of the menstrual cycle (*p* < 0.05). Bcl-2 was more expressed in UF from fertile women than from menopausal women (*p* < 0.05).
		Zhang et al. [64]	Comparison of Bcl-2 protein expression in UF and normal myometrium (*n* = 40).	Bcl-2 was highly expressed in UF during the whole menstrual cycle compared to NM (*p* < 0.01). The increase rate was higher in the secretory phase of the menstrual cycle than in the proliferative phase (*p* < 0.01).
		Kovács et al. [66]	Comparison of Bcl-2 protein in normal and UF specimens from cyclic (*n* = 16) and menopausal (*n* = 5) women.	The amount of Bcl-2 in the UF was higher than in the NM (*p* < 0.05) in the proliferative and secretory phases (*p* < 0.05). The rate of increase was higher in the secretory phase than in the proliferative phase. In menopausal women, no change was detected in UF relative to NM.
		Csatlós et al. [67]	Comparison of Bcl-2 mRNA in UF women (*n* = 101) and control women (*n* = 110).	Bcl-2 mRNA overexpression in UF compared to the control group (*p* = 0.02–0.04). Bcl-2 expression was positively correlated with the number of tumors.
		Fletcher et al. [39]	Comparison of Bcl-2/Bax RNA ratio in UF and NM cell lines exposed to normal (20% O2) and hypoxic (2% O2) conditions for 24 h.	Higher level of Bcl-2/Bax ratio in UF than in NM under normoxic conditions (*p* < 0.001). Hypoxia increased the Bcl-2/Bax ratio and decreased the level of apoptosis in UF; in contrast, changes in UF were opposite (decreased Bcl-2/Bax, *p* < 0.04).
CDKN1A	Regulator of cell cycle progression at the G1 phase.	Salimi et al. [68]	Comparison of *CDKN1A* 98A allele frequency in genomic DNA extracted from blood samples of 154 women with UF and 197 matched controls.	The frequency of the *CDKN1A* 98A allele was significantly higher in the UF women compared to controls (*p* = 0.04).

The table provides a summary of the world’s studies on the analysis of expression, as well as the analysis of structural variations in the genes involved in the HIF1 pathway. *—The factor is responsive to HIF according to KEGG pathway database. https://www.kegg.jp/pathway/hsa04066 (accessed on 25 October 2022). ** Functions of HIF-responsive elements were descripted due to the Gene-cards database. https://www.genecards.org (accessed on 2 February 2023).

**Table 2:** Overrepresented biological processes, reflecting genes encoding HIF subunits (*HIF1A*, *HIF1B* (*ARNT*), *HIF2A* (*EPAS1*), *HIF3A*) and their main functional partners (*ATM*, *CD44*, *CDC42*, *FOXO1*, *IGF1*, *RBMS1*, *SIM2*, *SIRT3*, *TERT*, *THRB*, *TP53*, *WT1*, *ZEB1*).

Biological Process	Fold Enrichment	FDR
Positive regulation of transcription from RNA polymerase II promoter in response to hypoxia	>100	2.04 × 10^−3^
Regulation of transcription from RNA polymerase II promoter in response to oxidative stress	>100	2.90 × 10^−3^
DNA damage response, signal transduction by P53 class mediator resulting in cell cycle arrest	>100	1.21 × 10^−2^
Positive regulation of nitric-oxide synthase activity	>100	1.37 × 10^−2^
Positive regulation of glycolytic process	>100	1.36 × 10^−2^
Regulation of DNA damage response, signal transduction by P53 class mediator	93.16	1.02 × 10^−3^
Regulation of intrinsic apoptotic signaling pathway by P53 class mediator	71.24	2.56 × 10^−2^
Positive regulation of vascular associated smooth muscle cell proliferation	65.47	2.87 × 10^−2^
Positive regulation of smooth muscle cell migration	65.47	2.86 × 10^−2^
Myoblast differentiation	62.11	3.02 × 10^−2^
Negative regulation of reactive oxygen species metabolic process	55.05	3.59 × 10^−2^
Positive regulation of cell growth	22.15	2.23 × 10^−2^
Mesenchymal cell differentiation	21.25	2.43 × 10^−2^
Negative regulation of apoptotic signaling pathway	21.15	3.97 × 10^−3^
Positive regulation of apoptotic process	9.50	3.83 × 10^−2^
Data were obtained using the bioinformatics tool Gene Ontology (http://geneontology.org/ (accessed on 4 June 2023))		

## Data Availability

The data presented in this study are available upon request from the corresponding author.

## Abbreviations

UF	uterine fibroids
NM	normal myometrium
HIF	hypoxia-inducible factor
EPO	erythropoietin
VHL	von Hippel–Lindau
CUL2	cullin 2
VEGF	vascular endothelial growing factor
KEGG	Kyoto Encyclopedia of Genes and Genomes
ANG-1	angiopoietin-1
VEGFR-1	vascular endothelial growing factor receptor 1
EGF	epidermal growing factor
SERPINE1 (PAI1)	plasminogen activator inhibitor 1
TEK	endothelial tyrosine kinase
TIMP1	tissue inhibitor of metalloproteinases 1
HMOX1	heme oxygenase 1
EDN1	endothelin 1
iNOS	inducible nitrogen oxide synthase
cNOS (eNOS)	constitutive (endothelial) nitric oxide synthase
GLUT-1	glucose transporter type 1
HK	hexokinase
ALDOA	aldolase, fructose-bisphosphate A
ENO1	enolase 1
PGK1	phosphoglycerate kinase 1
PFK/FBPase 3	6-phosphofructo-2-kinase/fructose-2,6-bisphosphatase 3
LDHA	lactate dehydrogenase A
HLRCC	hereditary leiomyomatosis and renal cell cancer
FH	fumarate hydratase
F2,6BP	fructose-2,6-bisphosphate
GWAS	genome-wide association studies
Bcl-2	B-cell lymphoma 2
STRING	Search Tool for the Retrieval of Interacting Genes/Proteins
ROS	reactive oxygen species
PPI	protein–protein interactions
ATM	ataxia telangiectasia mutated
CDC42	cell division control protein 42 homolog
FOXO1	forkhead box protein O1
IGF1	insulin-like growth factor I
RBMS1	RNA-binding motif, single-stranded-interacting protein 1
SIM2	single-minded homolog 2
SIRT3	NAD-dependent protein deacetylase sirtuin-3, mitochondrial
TERT	telomerase reverse transcriptase
THRB	thyroid hormone receptor beta
TP53	tumor protein P53
WT1	Wilms tumor 1
ZEB1	zinc finger e-box binding homeobox 1
